# Psychological Impact of the COVID-19 Pandemic on Athletes

**DOI:** 10.3389/fspor.2021.603415

**Published:** 2021-04-21

**Authors:** Clifford C. Uroh, Celina M. Adewunmi

**Affiliations:** ^1^Youth Sports Club, Akoka Study Centre, Lagos, Nigeria; ^2^Human Kinetics and Health Education, University of Lagos, Lagos, Nigeria

**Keywords:** psychological distress, viral pandemic, athletes, athletic identity, age

## Abstract

This study explored the influence of athletic identity and sports participation on the psychological well-being of athletes during a pandemic. The objective of the study was to understand the psychological impact of the coronavirus lockdown measure on athletes who were not able to carry out their normal daily routine. Athletes from nine different sports completed an online survey during the sixth week of the total lockdown in Nigeria. The online survey consists of an athletic identity scale and the Kessler Psychological Distress Scale (K10). Data collected were analyzed using Multiple regression and the Mann-Whitney test at 0.05 level of significance. The result of the findings revealed that category of sports (individual and team) (*M* = 0.73, *β* = −6.116) and athletic identity (*M* = 59.16, *β* = −0.166) predicts psychological distress to some degree. Few individual sports athletes and athletes with low athletic identity are prone to higher levels of psychological distress than team sports athletes and athletes with high athletic identity during the coronavirus pandemic lockdown. Individual sports athletes reported elevated levels of psychological distress compared to team sports athletes (*z* = −2.186, *p* = 0.03, *r* = 0.27). In conclusion, the results have confirmed that some athletes competing in individual sports experience elevated levels of psychological distress during the coronavirus pandemic, therefore they need the support of a sports psychologist during such periods to help in maintaining their psychological well-being.

## Introduction

Pandemics have been around for more than a century, and they continue to impact humanity negatively. There have been pandemics such as the H1N1 in Mexico, which rapidly spread to the whole world (Cowling et al., 2010), the MERS-COV, Ebola, and SARS which came later. These pandemics cause enormous negative economic, social, and security impacts on the global community (Qiu et al., 2017), but their psychological influence is hardly recognized. Recently, the world started to experience another pandemic caused by the novel coronavirus-19 that originated in Wuhan, China (Toresdahl and Asif, 2020). The coronavirus has a similar mode of transmission to SARS and MERS (Dong and Bouey, 2020), but it has spread so fast to many other countries, including Nigeria.

With the large-scale spread of this coronavirus and the knowledge of its transmission, most governments introduced heightened measures to control its spread (Taylor et al., 2008; Dong and Bouey, 2020). These measures include the use of protective equipment and the introduction of non-pharmaceutical protocols such as social distancing, hygiene guidelines, and, in more severe cases, a total lockdown (Cowling et al., 2010; Schinke et al., 2018). The consequence of these measures implied that gatherings of people were not welcome (Dong and Bouey, 2020), and as a result, many sports events earlier scheduled were cancelled or suspended due to the high risk of spreading the virus. The major events postponed include the Olympics slated to be held in Tokyo, UEFA Euro Cup, UEFA Champions League, and CAF Nations Cup qualifiers (Samuel et al., 2020). Most football leagues and competitions were also affected by safety measures. In Nigeria, the National Sports Festival was postponed indefinitely, pending advice from the Nigeria Centre for Disease Control (Okpara, 2020). As expected, the postponement of these competitions came with a lot of economic hardships on host cities and sports event organizers. Sports fans also had their fair share. These postponements also have a significant psychological influence on the athletes who are to participate in these competitions.

Most studies have focused on the psychological implication of pandemics on the general population (McAlonan et al., 2007; Taylor et al., 2008), with very few paying attention to special populations like athletes in recent times (Turbeville et al., 2006; Pillay et al., 2020). Recent research on home confinements as a result of the coronavirus have shown that athletes experience negative psychological effects (Dong and Bouey, 2020; Toresdahl and Asif, 2020). In fact, some studies have revealed that athletes experience mental health challenges just like the general population and are therefore not immune (Gouttebarge and Kerkhoffs, 2018; Schinke et al., 2018; Pillay et al., 2020). On the other hand, other studies have stated that sports provide a protective effect that prevents psychological disturbances. For example, a study was carried out to compare general health in athlete and non-athlete women (Alamdarloo et al., 2019). The study found out that athletes differed from non-athletes in anxiety and severe depression, with the mean scores for these two variables lower in athletes compared to non-athletes. Similarly, another study suggested that physical activity in the right quantity has the potential to reduce symptoms of anxiety and depression (Siefken et al., 2019). It seems that there is no agreement as to the existence of mental health problems among sports athletes due to the belief that sports prevent ill mental health. However, recent studies on mental health in sports among elite athletes have revealed that, compared to the general population, athletes are under-diagnosed, and the culture of mental toughness promotes this situation (Schinke et al., 2018). Many athletes who exhibit these ill mental health symptoms, such as distress, burnout, depression, or sleep disturbance, adverse feelings or thoughts, and drug abuse (Gouttebarge and Kerkhoffs, 2018), hide it from their teammates and coaches due to the mental toughness culture that pervades the atmosphere.

Furthermore, athletes who suffer from these conditions may not inform their coach or teammates for fear of losing their playing position. If they compete in individual sports, they may not want to be deprived of competing against an opponent. Studies have shown that individual sports athletes are at a greater risk of psychological distress than team sports athletes (Tasiemski and Brewer, 2011; Purcell et al., 2019). Mental health challenges have been suggested to be more prevalent in individual sports athletes compared to team sport athletes (Pluhar et al., 2019), owing to the nature of individual sports. Specifically, individual sports athletes have to deal with both success and failure on their own, while team sports athletes enjoy a shared responsibility (Mladenović, 2019). Furthermore, team sports provide more social opportunities compared to individual sports in which there is no peer support. This situation may be tougher for athletes who are much younger and inexperienced (Nicholls et al., 2016). For instance, studies have emphasized the high risk which college athletes are exposed to, including the inability of college athletes in dealing with the challenges that they encounter. In fact, youth athletes who are unable to deal with perceived failure prefer to drop out of sports participation (Crane and Temple, 2015), hence experiencing a depletion in their athletic identity. Similarly, a study on collegiate athletes' reaction to loss revealed that unsatisfied athletes tended to decrease their athletic identity compared to athletes who were satisfied by their performance (Brewer et al., 1999). Athletes reduced their self-identification with their athletic role as a response to poor performance.

Athletic identity refers to the degree to which an individual attributes or identifies with the athletic role. Although it is developed through social interaction and validation, it is accompanied by both negative and positive outcomes (Verkooijen et al., 2012; Reifsteck, 2013). This identity with the athlete's role is confirmed by others and motivates the individual involved to be more committed to training and sports goal achievement. Athletes who are high in athletic identity exhibit behaviors such as going regularly to the gym and sports field and even buying mostly athletic gears and equipment. However, an overemphasis on athletic identity may lead to negative outcomes as stated earlier. Studies have stated that a reduction in the opportunity to participate in sports harms the mental health of athletes and their athletic identity levels (Masten et al., 2006; Miller and Hoffman, 2009). Consequently, when an athlete is not regularly allowed to express himself/herself, there is a high tendency for that athlete to experience a reduced athletic identity (Fraser-Thomas et al., 2008; Altintaş and Kelecek, 2017). This phenomenon is expected more in youth athletes, who may drop out of sports (Nicholls and Polman, 2007; Hall et al., 2017; Lewis et al., 2017), than in elite professional athletes who may cope with such a situation. In addition, it has also been revealed in other studies that when athletes, especially those high in athletic identity, are unable to engage in their daily routine as a result of injury (Mittly and Nemeth, 2016), they start to resent their identification as athletes (Hadiyan and Sheikh, 2015). This makes athletic identity a critical factor that should be considered especially in athletes as they go through the period of home confinement during the coronavirus pandemic.

Although recent studies are beginning to investigate the psychological well-being of athletes during the coronavirus pandemic (Costa et al., 2020; Mehrsafar et al., 2020; Pillay et al., 2020), most of these studies are from Europe and the USA. More studies are required to better understand athletes' experience from other climes like Africa and, specifically, Nigeria. There is limited knowledge about the influence of athletic identity and sports participation on the psychological well-being of athletes, especially during a pandemic. A recent study explored the differences in gender, type of sport, and competitive level in athletic identity during the coronavirus lockdown and found out that elite athletes and team sports athletes showed higher athletic identity (Costa et al., 2020). However, this study did not investigate the influence of extrinsic rewards on psychological well-being and how athletic identity interacts with well-being in athletes during the coronavirus lockdown. Based on this, our study aims to investigate differences by competitive level, extrinsic rewards, and category of sports participation in athletes and understand how athletic identity, age, category of sport participation, and the number of years participating in sports interact with the psychological well-being of athletes during the coronavirus lockdown.

## Methodology

### Participants and Procedure

The sample comprised 64 athletes from nine different sports: two team sports (football and basketball) and seven individual sports (athletics, cycling, taekwondo, tennis, gymnastics, badminton, and table tennis). The participants were classified as professional athletes (*n* = 20), which included athletes who compete in national and regional tournaments, and non-professional athletes (*n* = 44) who compete in state tournaments. The authors contacted coaches known to them through voice calls and WhatsApp messages to help in reaching their athletes about the possibility of taking part in the study. This was done during the home confinement period of the total lockdown which started at the end of March 2020. A weblink to the consent form and online survey was made available to coaches and some athletes *via* WhatsApp from May 5, 2020, during the height of the pandemic lockdown in Nigeria. The online survey was open for 4 weeks from the May 5, 2020 to June 2, 2020. Due to the circumstances at that period, the authors could not determine which sports athletes could take part in the study. Hence, those who responded to the survey by completing it were regarded as study participants, and the participation was anonymous. Since we were interested in athletes all over the country, we did not request their location. The ethical approval board of the Department of Human Kinetics and Health Education was contacted by email, and they approved the study.

### Measure

The full survey comprised 26 questions covering a range of subject areas; those reported here include demographic information: gender, age category, type of sport, and level in sport. In addition, the respondents were asked about their years of sports participation as well as earnings from sports.

#### Anxiety and Depression During the Viral Pandemic

To measure psychological distress during the pandemic, the 10 items from the Kessler Psychological Distress Scale (K10) were employed (Andrews and Slade, 2001; Kessler et al., 2002; Sampasa-Kanyinga et al., 2018). The K-10 was developed and validated as a screening tool for assessing the likelihood of common mental disorders in the general population and clinical samples (Kessler et al., 2002; Slade et al., 2011; Sunderland et al., 2012). The K-10 demonstrates strong psychometric properties (Pereira et al., 2019) and has been used across different populations and cultures (Chan and Fung, 2014; Sampasa-Kanyinga et al., 2018). The K10 is a 10-item scale that asks respondents how often they have experienced certain symptoms during the preceding 4 weeks. The participants responded on a five-point scale depending on how frequently they experienced each symptom. The five-point scale ranges from none of the time (1) to all of the time (5). The K10 has items such as item 1 (In the past 4 weeks, about how often did you feel tired out for no good reason?) and item 5 (In the past 4 weeks, about how often did you feel restless or fidgety?). Past research has shown that K10 has satisfactory psychometric properties (Taylor et al., 2008; Sampasa-Kanyinga et al., 2018). In this study, the internal consistency was satisfactory (α = 0.86).

#### Athletic Identity

To measure athletic identity, the original 10-item scale for athletic identity measurement was used (Brewer et al., 1993; Hadiyan and Sheikh, 2015; Tunçkol, 2015). This test was chosen because of its ability to measure levels of athletic identity and its high internal consistency index test–retest reliability. The Athletic Identity Measurement Scale is supported as a unidimensional and multidimensional instrument (Brewer and Cornelius, 2001). The evaluation of the unidimensional scale can be performed by using the total score to produce a single self-evaluation score that represents the athletic identity, with higher scores indicating a strong athletic identity. The multidimensional scale contains four scales: self-identity, social identity, exclusivity, and negative affectivity (Brewer et al., 1993). Self-identity items capture reports that are self-referenced. Social identity items express the degree to which individuals view themselves as occupying the athletic role. Exclusivity measures the self-worth of an individual established through participation in the athletic role. Negative affectivity is the degree to which individuals experience negative emotions from undesirable sporting outcomes such as injury or retirement. The participants were asked to indicate their agreement with each item by responding on a seven-point scale from “strongly agree” to “strongly disagree.” The scale comprised 10 items measuring self-identity (e.g., “I have many goals related to sport”), social identity (e.g., “Most of my friends are athletes”), negative affectivity (e.g., “I feel bad about myself when I do poorly in sport”), and exclusivity (e.g., “Sport is the most important part of my life”). The internal consistency of this questionnaire was satisfactory (α = 0.93).

### Data Analysis

The characteristics of the participants were described using frequency distribution, while group differences were subjected to Mann–Whitney tests since the data collected did not meet the assumptions of parametric tests such as small samples and normality of data. To understand whether one group experiences psychological distress more than the other during the coronavirus lockdown, the Mann–Whitney test was conducted to test the difference between professional and non-professional athletes, team and individual sports athletes, and athletes who earned financial rewards from sports participation and athletes who do not earn from sports participation. Multiple regression analysis was performed in order to determine the possible effect of the interaction among athletic identity, years participating in sports, age, and psychological distress scores (criterion variable). We used STATA version 14 for all statistical analysis. All statistical tests were performed at 0.05 level of significance.

## Results

Sample characteristics are displayed in Tables 1 and 2. Results from the Mann–Whitney tests are shown in Table 3. Analysis by competitive level (non-professional and professional) did not yield any significant difference on psychological well-being between professional and non-professional athletes (*z* = 0.63, *p* = 0.53, *r* = 0.08). Specifically, professional athletes were not different from non-professional athletes based on psychological distress. There was a significant difference between individual sports athletes and team sports athletes (*z* = −2.19, *p* = 0.03, *r* = 0.27), but with a small effect size. Financial opportunities did not reveal any significant difference. Athletes who earned from their participation in sports did not differ significantly in psychological distress from those who did not earn from sports participation (*z* = 0.46, *p* = 0.65), and the effect size of the analysis was small (*r* = 0.06).

**Table 1:** Sample description.

		***n***	**Percentage (%)**
Gender (*n* = 65)	Male	56	88
	Female	8	13
Age category	13–17	7	11
	18–23	37	58
	24–29	17	27
	30–35	3	5
Earn from sports participation	No	27	42
	Yes	37	58
Type of sports	Athletics	2	3
	Badminton	7	11
	Basketball	3	5
	Cycling	1	2
	Football	44	69
	Gymnastics	2	3
	Table tennis	1	2
	Taekwondo	2	3
	Tennis	2	3
Level in sport	Professional	20	31
	Non-professional	44	69
Years in sports participation	1–5 years	19	30
	6–10 years	19	30
	11–15 years	11	17
	Over 15 years	15	23

**Table 2:** Prevalence of psychological distress by sample characteristics.

		**Low (10–19)** **(%)**	**Moderate (20–24)** **(%)**	**High (25–29)** **(%)**	**Very High (30–50)** **(%)**
Gender	Male	54	14	18	14
	Female	29	0	29	43
Earn from sports participation	No	50	19	19	12
	Yes	47	8	19	25
Category in sport	Individual sports	31	13	19	38
	Team sports	57	13	20	11
Level in sport	Professional	53	16	16	16
	Non-professional	47	12	21	21

**Table 3:** Mann-Whitney tests for psychological distress.

**Variables**		***M***	***SD***	***p*-Values**
Level in sports	Professionals	20.40	9.08	0.527
	Non-professionals	21.27	8.29	
Category of sport	Team sports athletes	19.34	7.29	0.029^*^
	Individual sports athletes	25.59	9.99	
Financial difference in sports participation	Athletes who earn	21.43	8.83	0.648
	Athletes who do not earn	20.41	8.11	

* *P < 0.05*.

Results from the multiple regression with psychological distress as the dependent variable and athletic identity, age, sport type, and years participating in sports as predictor variables are displayed in Table 4. The results show that the categories of sport participation (*b* = −6.116, *SE* = 2.610, *p* = 0.023) and athletic identity (*b* = −0.166, *SE* = 0.075, *p* = 0.03) have significant but negative relationships with psychological distress. The other variables had no significant relationship with psychological distress.

**Table 4:** Regression result for psychological distress.

	***M***	***SE***	***β***
Constant		7.164	0.0001
Athletic identity	59.16	0.073	−0.143^*^
Sport type	0.73	2.610	−6.116^*^
Age	21.86	0.261	−0.438
Years participating in sports	9.66	0.224	−0.058
R-squared	0.20		
No. of observations	64		

* *P < 0.05*.

## Discussion

This study sought to investigate the influence of athletic identity on the psychological well-being of athletes during the COVID-19 pandemic. In this study, we were interested in finding out the differences in psychological distress between professional and non-professional athletes, between individual and team sports athletes, and between athletes who earned from sports participation and those who do not earn from sports participation during the COVID-19 pandemic lockdown. There were two significant findings of this study. The first is that individual athletes differed significantly from team sports athletes in their psychological response to the COVID-19 pandemic lockdown. Secondly, we found out that athletic identity has a significant relationship with psychological distress.

Our study revealed that individual sports athletes experienced high psychological distress compared to team sports athletes who experienced low psychological distress. However, our result should be interpreted with caution, owing to the number of individual athletes who participated in the study. Individual sports athletes are at a higher risk of experiencing psychological distress due to the pandemic lockdown that prevents participation in regular sporting activities. Studies have shown that individual sports athletes are at a greater risk of psychological distress than team sports athletes (Tasiemski and Brewer, 2011; Purcell et al., 2019). Furthermore, other studies have suggested that individual sports provide little or no social opportunities and a lot of personal responsibility for both success and failure, hence making individual athletes more prone to psychological distress compared to team sports athletes (Dias et al., 2010; Mladenović, 2019; Pluhar et al., 2019). For instance, individual sports athletes attribute failure to themselves more than athletes in team sports where there is a diffusion of responsibility, and this situation makes them more prone to psychological distress when compared to team sports athletes (Nixdorf et al., 2016).

Based on the result from the multiple regression analysis, our study showed that the age of athletes and the number of years spent participating in sports did not predict psychological distress. However, athletic identity and category of sport participation were able to predict psychological distress to some degree. Athletes who competed in individual sports experienced higher psychological distress compared to those who competed in team sports. This finding is supported by a number of studies (Dias et al., 2010; Pluhar et al., 2019). The circumstances surrounding the nature of individual sports make the athletes who take part in individual sports prone to psychological distress than team sports athletes (Nixdorf et al., 2016; Purcell et al., 2019). Furthermore, athletes who reported higher athletic identity experienced lower psychological distress compared to those who reported lower athletic identity. Although there are no studies that have directly reported this finding, our findings are similar to a previous study which showed that an increase in self-identity decreases anxiety levels (Masten et al., 2006). The abrupt end to sports events and activities brought about by the COVID-19 lockdown is associated with a loss of aspects that contribute to one's sense of self, which can negatively affect self-identity and lead to depressive symptoms (Tasiemski and Brewer, 2011).

We found out that both professional and non-professional athletes do not differ in the symptoms of psychological distress that they reported. This result has been confirmed in a study that sought to investigate the effect of soccer on mental health (Heun and Pringle, 2018). It was concluded that participants in football are not different from the general population in mental health problems. Specifically, there are general risk indicators such as negative life events in which both athletes and the general population experience comparable psychological distress (Rice et al., 2016; Purcell et al., 2019).

With regards to financial compensation, there was no significant difference in psychological distress between athletes who earned from sports participation and other athletes who did earn from their participation in sports. Previous studies have highlighted the importance of non-monetary rewards to athletes over monetary rewards (Podlog et al., 2015; Maier et al., 2016). Hence, our study suggests that the absence of extrinsic rewards from sports during the COVID-19 pandemic lockdown did not influence the psychological reaction of athletes who usually received financial reinforcement.

The findings of this study have implications for coaches and athletes who compete at state, regional, and national sports tournaments in Nigeria. Nigerian athletes, especially individual sports athletes who depend largely on their sport as a means of achieving their goals, may suffer the consequences of this over-dependence during the COVID-19 lockdown. Sports psychologists are not usually present as part of the support staff to athletes in most sports federations. For this reason, local sports coaches work with athletes based on their experience alone, without any knowledge of mental health, thereby increasing the chances of mental health problems in athletes. Nigeria sports federations should engage the services of sports psychologists as part of the support staff to athletes, include courses that emphasize psychological education in the training programs of coaches, and organize online mental health seminars as part of athlete education during and beyond the lockdown period.

## Limitations

The main limitation of this study was the size of the sample, which is relatively low and had more respondents from one sport (football). In addition, most of our samples were from team sports. Hence, the data cannot be easily generalized. More studies with larger samples are needed to be able to generalize the findings. Our study was not able to compare gender differences due to the low number of female athletes responding; more studies are required in this regard. Furthermore, the study included a self-report psychological distress scale which was not validated for the intended population. Hence, we cannot draw any causal conclusion based on the current findings.

## Conclusion

In conclusion, the results of this study indicate that individual sports athletes experienced high psychological distress as a result of the COVID-19 pandemic lockdown. The number of individual sports participants in the study were fewer compared to team sports participants, and this may have contributed to this result. Hence, this result should be interpreted with caution. Furthermore, this result should inform practitioners to pay closer attention to athletes who compete in individual sports. For example, the COVID-19 pandemic lockdown is a peculiar situation in which athletes had to be in isolation, away from their sport without any certainty of resumption or any form of real social support from relatives and friends. It might have led to feelings of sadness and hopelessness in some individual sports athletes. For this reason, coaches and the other staff who work with individual sports athletes should regularly keep in touch with the athletes using online interventions during the pandemic lockdown to help them have a sense of belonging. Further research should focus on determining, by comparing different sports, the highest prevalence of psychological distress.

Our data showed that athletic identity and category of sports participation predict, to a certain extent, the level of psychological distress athletes experience during the COVID-19 pandemic lockdown. These findings may have important implications for practitioners, coaches, and athletes. A number of studies have suggested that individual sports athletes experience anxiety and psychological distress more than team sports athletes (Pluhar et al., 2019). With regards to athletic identity, it is important to note that athletes may reduce their connection with the athletic role as a means of protecting their self-image (Brewer et al., 2010). When some athletes begin to divest or reduce their athletic identity during a pandemic, it may be a sign that they are experiencing psychological distress. However, this might not be the case for other athletes. Nevertheless, it demands some level of awareness from coaches and sports psychologists. Hence, athletic identity should be considered not as the cause of psychological distress but as a potential predictor of psychological distress during pandemic lockdowns. Sports federations in Nigeria should employ sports psychologists who can support athletes in readjusting their goals and adapting to changing circumstances like the coronavirus pandemic lockdown, using accessible online interventions. More research is needed to understand the interaction between athletic identity and psychological distress.

## Data Availability Statement

The raw data supporting the conclusions of this article will be made available by the authors, without undue reservation.

## Ethics Statement

The studies involving human participants were reviewed and approved by Department of Human Kinetics and Health Education. The patients/participants provided their written informed consent to participate in this study.

## Author Contributions

CU and CA wrote the Introduction, discussion and conlusions. Data collection and analysis was mainly done by CU. The authors approved the submitted version.

## Conflict of Interest

The authors declare that the research was conducted in the absence of any commercial or financial relationships that could be construed as a potential conflict of interest.
